# Gender Specific Re-organization of Resting-State Networks in Older Age

**DOI:** 10.3389/fnagi.2016.00285

**Published:** 2016-11-25

**Authors:** Aimée Goldstone, Stephen D. Mayhew, Izabela Przezdzik, Rebecca S. Wilson, Joanne R. Hale, Andrew P. Bagshaw

**Affiliations:** ^1^Birmingham University Imaging Centre (BUIC), School of Psychology, University of BirminghamBirmingham, UK; ^2^Center for Health Sciences, SRI International, Menlo ParkCA, USA; ^3^Department of Cognitive Neuroscience, Radboud University Medical CentreNijmegen, Netherlands

**Keywords:** ACC, aging, gender, functional connectivity, inter-network, intra-network

## Abstract

Advancing age is commonly associated with changes in both brain structure and function. Recently, the suggestion that alterations in brain connectivity may drive disruption in cognitive abilities with age has been investigated. However, the interaction between the effects of age and gender on the re-organization of resting-state networks is not fully understood. This study sought to investigate the effect of both age and gender on intra- and inter-network functional connectivity (FC) and the extent to which resting-state network (RSN) node definition may alter with older age. We obtained resting-state functional magnetic resonance images from younger (*n* = 20) and older (*n* = 20) adults and assessed the FC of three main cortical networks: default mode (DMN), dorsal attention (DAN), and saliency (SN). Older adults exhibited reduced DMN intra-network FC and increased inter-network FC between the anterior cingulate cortex (ACC) and nodes of the DAN, in comparison to younger participants. Furthermore, this increase in ACC-DAN inter-network FC with age was driven largely by male participants. However, further analyses suggested that the spatial location of ACC, bilateral anterior insula and orbitofrontal cortex RSN nodes changed with older age and that age-related gender differences in FC may reflect spatial re-organization rather than increases or decreases in FC strength alone. These differences in both the FC and spatial distribution of RSNs between younger and older adults provide evidence of re-organization of fundamental brain networks with age, which is modulated by gender. These results highlight the need to further investigate changes in both intra- and inter-network FC with age, whilst also exploring the modifying effect of gender. They also emphasize the difficulties in directly comparing the FC of RSN nodes between groups and suggest that caution should be taken when using the same RSN node definitions for different age or patient groups to investigate FC.

## Introduction

Advancing age is associated with a wide range of changes to human brain structure and function (Raz and Rodrigue, 2006) which includes an annual tissue loss of approximately 2.1% between the ages of 70 and 80 years (Tang et al., 2001) and declines in both gray (Raz et al., 1997; Sowell et al., 2003; Tisserand et al., 2004) and white (Resnick et al., 2000, 2003; Bartzokis, 2004) matter. White matter integrity and patterns of functional connectivity (FC) are strongly associated (Andrews-Hanna et al., 2007; Chen et al., 2009; Damoiseaux and Greicius, 2009; Hermundstad et al., 2013), and recent research has begun to investigate changes in FC in the older brain (see Ferreira and Busatto, 2013, for a comprehensive review). The term ‘FC’ can broadly be defined as the statistical association or dependency between the blood oxygenation level-dependent (BOLD) signal time-series of anatomically distinct brain regions (Friston et al., 1996; Horwitz, 2003; Friston, 2011). This approach has lead to the identification of a series of resting-state networks (RSNs; Fox et al., 2005; Di and Biswal, 2013), which are thought to support cognitive functions (Stevens and Spreng, 2014). These include the default mode (DMN), dorsal attention (DAN), saliency (SN), visual, motor, and auditory networks. The DMN, which comprises the posterior cingulate cortex (PCC), medial prefrontal cortex (mPFC), inferior parietal lobes (IPL), and the medial temporal lobes (MTL) (Buckner et al., 2008; Andrews-Hanna et al., 2010), has been the most commonly studied RSN in relation to age. Numerous studies have reported decreased FC within the DMN in older compared to younger adults (Andrews-Hanna et al., 2007; Wu et al., 2011; Tomasi and Volkow, 2012). In addition, Damoiseaux et al. (2008) reported that out of a number of RSNs, only the DMN showed decreases in FC with age, suggesting that this network is particularly susceptible to aging effects. However, more recently, others have reported age-related FC decreases in the SN (Onoda et al., 2012), motor network (Wu et al., 2007), and visual network (Yan et al., 2011).

Although the majority of resting-state studies have found decreases in intra-network FC with advancing age, there have been several instances where increases in intra-network FC have been identified (Toussaint et al., 2011; Mowinckel et al., 2012). While decreases in FC with age are often interpreted as indicators of reduced processing ability of the networks involved, the interpretation of increases in FC with age is currently less clear and could be related to changes in neurotransmitters, vasculature, or compensatory network re-organization in response to a decline in the FC of critical networks (Riddle et al., 2003; Peters, 2006). However, although greater FC may represent a stronger communication exchange or a more efficient network in a variety of situations, segregation of the activity of particular brain regions is likely to be just as important for efficient cognitive performance.

Investigation of inter-network FC may also enhance our understanding of how the brain alters with age. By investigating how nodes of a particular network become more or less connected to nodes of a separate network we may be able to gain a greater insight into the network re-organization that potentially occurs with advancing age. To date, few studies have focussed their attention on inter-network FC, although there is some evidence that inter-network FC is altered with age (Onoda et al., 2012; Tomasi and Volkow, 2012). He et al. (2013) demonstrated that inter-network FC between the right insula and nodes of the DMN and Central Executive Network (CEN) was significantly reduced with age. In contrast, others have suggested that while intra-network FC is often reduced in older age, inter-network FC is often increased, suggesting a more complex picture of connectivity alterations in the aging brain (Betzel et al., 2014; Geerligs et al., 2014b). Similar findings have been reported during task-based FC analysis (Voss et al., 2010; Geerligs et al., 2014a). Taken together these recent findings seem to suggest that older age may be associated with reduced specificity of RSNs, which become less modular and distinct and more inter-connected and diffusely distributed across the brain as we age.

As well as the influence of advancing age on the brain, it is well established that there are gender differences in brain structure, chemistry, and function (Cosgrove et al., 2007; Luders et al., 2009; Ingalhalikar et al., 2014; Ruigrok et al., 2014). Significant age^∗^gender interactions have been identified in relation to brain structure across the lifespan (Raz et al., 1997; Gur et al., 2002; Riello et al., 2005; Sowell et al., 2007; Nunnemann et al., 2009). However, studies have also failed to find such interactions (Salat et al., 2004; Lemaitre et al., 2005; Greenberg et al., 2008) and a recent study reported that controlling for brain size resulted in a substantial decrease in the effect of gender (and gender^∗^age interactions) on brain volume (Jäncke et al., 2015). Given these differences in brain structure associated with gender, it is likely that gender also influences brain function. A number of studies have identified such differences (Biswal et al., 2010; Allen et al., 2011; Filippi et al., 2013; Smith et al., 2014). Furthermore, gender has also been found to modulate the lateralization of RSNs (Liu et al., 2009; Agcaoglu et al., 2015), although others have reported that gender has a relatively small (Bluhm et al., 2008; Lopez-Larson et al., 2011) or lack of effect (Weissman-Fogel et al., 2010; Nielsen et al., 2013) on RSNs. Studies that have investigated the effects of both advancing age and gender on FC have largely been limited to investigation of lateralization of RSNs, rather than specific effects on inter- and intra-network FC.

It is clear that both age and gender modulate patterns of resting-state FC; however, few studies have included gender when investigating how age may modulate intra- and inter-network FC. Furthermore, to date, no studies have specifically investigated whether the spatial location of RSN nodes differs with older age. Considering that age-related differences in gray matter are not homogeneous across the brain (Raz et al., 1997; Sowell et al., 2003; Tisserand et al., 2004), it is plausible that the spatial extent of particular RSN nodes, or indeed their location, may alter with age. It thus remains to be seen whether age-related FC differences are driven by changes to the connections between nodes or changes to RSN definitions, or both.

Our study aimed to further investigate the interactions between age and gender on both intra- and inter-network FC of the DMN, DAN, and SN. For this, we investigated FC using (1) the same node definitions for the two age groups (defined using data from an independent sample of participants) and (2) nodes defined separately for the two groups. We focussed our analysis on these RSNs as advancing age has been associated with disrupted FC of both the DAN and DMN (Andrews-Hanna et al., 2007; Zhang et al., 2014), while the SN is thought to be responsible for switching between the DMN and task-positive networks (Sridharan et al., 2008) and has also been shown to be affected by age (Onoda et al., 2012; He et al., 2013). While this approach may not capture all possible changes in FC associated with age and gender, we were motivated in our analysis by the benefits in terms of interpretability and comparability with previous studies. We concentrated on these ‘cognitive’ RSNs as the primary behavioral changes that occur with age tend to be in higher order cognitive functions, rather than basic sensory processing. However, we also present the results of the sensory RSNs in the Supplementary Material for completeness See Supplementary Figure S1.

## Materials and Methods

### Participants

Twenty younger (*M* = 27, ±3 years, 10 male) and twenty older (*M* = 74, ±4 years, 9 male) participants took part. Older participants were screened for cognitive impairment with the Advanced Mini-Mental State Test (3MS) (Teng and Chui, 1987); the group’s average score was 97.65 (±2.6, range: 88–100). No participants scored below the cut-off (79/100) for normal cognitive ability. All participants (excluding two younger participants for whom English was not their native language) also took part in the National Adult Reading Test (NART) as an estimator of IQ (Nelson and Willison, 1991). Younger participants had an average ‘full IQ’ score of 114 (±7.56), compared to a score of 119 (±7.07) for the older participants. IQ scores were not significantly different for the two groups, as assessed by a one-way ANOVA [*F*(1,37) = 3.811, *p* = 0.059].

### Procedure

Participants gave written informed consent and the study was approved by the Research Ethics Board of the University of Birmingham. All participants were screened for MR compliance and took part in the NART. In addition, all participants also completed the Epworth Sleepiness Scale (ESS) (Johns, 1991), which is a subjective measure of their propensity to fall asleep under different conditions and resulted in a ‘daytime sleepiness’ score for each participant. Older participants also completed the 3MS. Participants then underwent the MRI session. During the resting-state scan participants were asked to keep their eyes open and think of nothing in particular. Immediately after the resting-state scan, participants subjectively rated their sleepiness using the Karolinska Sleepiness Scale (KSS) (Shahid et al., 2012), and were asked to report if they were aware of falling asleep during the scan. Following the session participants were thanked and debriefed.

### MRI Procedure

A Philips Achieva 3T MR scanner with a 32-channel head coil was used to acquire MRI data. A 15 min resting-state scan was acquired (T2^∗^-weighted fMRI data with whole brain coverage: 3 mm × 3 mm × 4 mm voxels, TR = 2000 ms, TE = 35 ms, SENSE factor = 2, flip angle = 80°, volumes = 450). In addition, a high-resolution (1 mm isotropic) T_1_-weighted anatomical image was also obtained. During the resting-state scan, participant’s cardiac and respiratory cycles were measured using pneumatic bellows and a pulse oximeter. Foam padding was positioned around the head to reduce motion artifacts.

### Definition of Network Nodes

**FIGURE 1:**
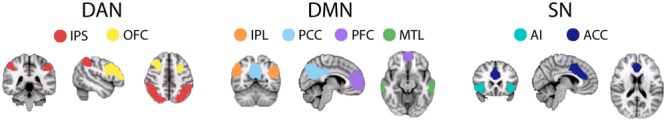
**Illustration of each node, defined from an independent, young cohort, of the three main ‘cognitive’ RSNs: DAN, DMN, and SN.** DAN comprises: IPS, intraparietal sulcus; OFC, orbitofrontal cortex; IPL, intraparietal lobe. DMN comprises: IPL, intraparietal lobe; PFC, pre-frontal cortex; MTL, medial temporal lobe. SN comprises: AI, anterior insula; ACC, anterior-cingulate cortex. For each node a 5×5×5 voxel ROI, created around the maximum Z-statistic voxel, was created for FC analysis.

The spatial locations of each RSN’s individual nodes were defined from 6-min resting-state scans (3 mm × 3 mm × 4 mm voxels, TR = 2000 ms, TE = 35 ms, flip angle 80°, SENSE factor = 2) acquired from an independent cohort of 55 subjects (28 male, age 25 ± 4 years) which was collected as part of a previous study (Przezdzik et al., 2013). Using FSL 4.1.8 ^1^ data were motion corrected, spatially smoothed (5 mm), temporally concatenated across subjects and decomposed into 20 spatially independent components using MELODIC (Beckmann and Smith, 2004). The DAN, DMN, and SN were visually identified from individual components, based on their spatial similarity to previous reports (Damoiseaux et al., 2006). Each component was thresholded at a Z-statistic >4, based on previous methodology (Khalsa et al., 2013), to ensure that each of the network nodes were spatially distinct. Each network was then manually separated into its individual nodes, these consisted of: left and right orbitofrontal cortex (OFC), left and right intraparietal sulcus (IPS) (DAN), mPFC, PCC, left and right IPL, left and right MTL (DMN), left and right insula and anterior cingulate cortex (ACC) (SN) (**Figure 1**).

### ROI Definition

FLIRT (Jenkinson et al., 2002) was then used to transform these node masks into functional space, using the T_1_ as an intermediate step, for each participant. For each RSN node a 5 × 5 × 5 voxel cube ROI, centered on the maximum Z-statistic voxel, was defined (**Table 1**). This resulted in an ROI of 125 voxels in size, for each RSN node. Before these ROIs were used to calculate FC we first accounted for differences in the proportion of gray/white matter voxels within ROIs between the two age groups, FAST (Zhang et al., 2001) was used to segment each individual’s T_1_ image into gray matter, white matter and CSF. These partial volume maps were then transformed into functional space using FLIRT (Jenkinson and Smith, 2001; Jenkinson et al., 2002), with nearest neighbor interpolation and a threshold of 0.5 to preserve approximately the size of the original partial volume map. Using this map, only gray matter voxels within each ROI were included for FC analysis. **Table 2** displays the average final ROI sizes for the two age groups, after including only gray matter voxels, which were used for FC analysis. A mixed design ANOVA with the main effects age and node and the interaction term age^∗^node revealed that for all nodes, aside from left IPL, older adults had significantly fewer voxels within each ROI, after excluding white matter/CSF voxels, as indicated by a significant main effect of age [*F*(1,38) = 107.68, *p* < 0.001, η^2^= 0.74] and a significant age^∗^node interaction, following a mixed design ANOVA [*F*(7.87,96.65) = 9.6, *p* < 0.001, η^2^= 0.20]. See **Table 2** for the results from pairwise comparisons. However, ROI size was not found to differentially affect FC strength, for either age group, providing reassurance that any differences in FC between the two age groups was not driven by differences in ROI size. See Supplementary Figures S2a–d.

**Table 1:** MNI co-ordinates of the peak voxel for each RSN node, around which 5×5×5 voxel ROIs were created, defined using: (1) an independent cohort of 55 young participants (aged 25 ± 4 years); and (2) only the older participant’s data.

	Independent cohort			Older gICA			Difference		
	*x*	*y*	*z*	*x*	*y*	*z*	*x*	*y*	*z*
**DAN**									
Left IPS	67	39	60	64	36	60	-3	-3	0
Right IPS	25	37	61	29	37	58	4	0	-3
Left OFC	71	75	52	67	84	48	-4	9	-4
Right OFC	21	83	45	22	70	52	1	-13	7
**DMN**									
PCC	45	37	53	45	35	52	0	-2	-1
mPFC	45	89	39	45	92	43	0	3	4
Left IPL	71	29	55	70	30	51	-1	1	-4
Right IPL	19	29	55	21	30	53	2	1	-2
Left MTL	77	58	27	75	62	28	-2	4	1
Right MTL	19	64	21	14	63	25	-5	-1	-4
**SN**									
ACC	45	76	51	43	70	59	-2	6	8
Left AI	65	71	37	73	66	39	8	-5	2
Right AI	27	75	37	26	68	38	-1	-7	1

*The final column reports the difference between the two sets of co-ordinates, for each direction.*

**Table 2:** Final ROI size (group mean number of voxels and standard deviation across participants) after transforming ROIs into individual space and selecting only gray matter voxels.

	Average ROI size			
	Younger	Older	Older (re-defined)	η^2^
**DAN**				
Left IPS	59 ± 8.81	49 ± 6.35 ^∗∗∗^	48 ± 6.95 ^∗∗∗^	0.33
Right IPS	61 ± 5.94	53 ± 6.14 ^∗∗^	42 ± 7.55 ^∗∗∗^	0.58
Left OFC	59 ± 6.83	51 ± 8.06 ^∗∗^	54 ± 8.21	0.15
Right OFC	62 ± 5.81	55 ± 9.50 ^∗∗^	53 ± 6.20 ^∗∗^	0.23
**DMN**				
PCC	79 ± 7.18	67 ± 8.76 ^∗∗∗^	70 ± 7.67 ^∗∗^	0.28
mPFC	85 ± 6.45	66 ± 8.74 ^∗∗∗^	55 ± 9.55 ^∗∗∗^	0.68
Left IPL	53 ± 10.14	49 ± 9.63	59 ± 8.34	0.18
Right IPL	47 ± 8.37	38 ± 7.17 ^∗∗^	58 ± 6.91 ^∗∗∗^	0.53
Left MTL	69 ± 7.04	52 ± 13.69 ^∗∗∗^	53 ± 11.90 ^∗∗∗^	0.31
Right MTL	68 ± 6.57	52 ± 12.68 ^∗∗∗^	43 ± 12.79 ^∗∗∗^	0.46
**SN**				
ACC	75 ± 6.91	42 ± 12.73 ^∗∗∗^	52 ± 10.29 ^∗∗∗^	0.70
Left AI	83 ± 7.52	61 ± 7.49 ^∗∗∗^	57 ± 7.04 ^∗∗∗^	0.68
Right AI	85 ± 7.51	65 ± 10.06 ^∗∗∗^	62 ± 9.51 ^∗∗^	0.65

**FIGURE 2:**
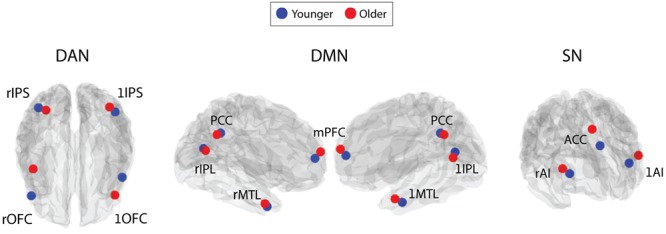
**Depiction of the peak z-statistic voxel location, around which 5×5×5 voxel ROIs were made, for each RSN node.** These were defined from gICAs performed on: an independent cohort of 55 younger participants (blue); and 20 older participants (red). L and R prefixed before node names indicates the left and right hemispheres. MNI co-ordinates for these voxels are presented in **Table 1**.

*Average ROI sizes are reported for the nodes defined using an independent cohort of 55 young participants (columns two and three) and only the older participant’s data (column four). Significant differences in ROI size between young and older adults are highlighted (^∗∗∗^*p* < 0.001, ^∗∗^*p* < 0.01). Partial eta squared (η^*2*^) is also displayed for each significant comparison. Spatial location of ROI peak voxels for young and older definitions are plotted in **Figure 2**.*

### Functional Connectivity Analysis

The effect of respiratory and cardiac confounds (RETROICOR) (Glover et al., 2000) and subsequently variations in breathing depth and heart rate interval (Birn et al., 2006; Chang et al., 2009) were reduced using custom MATLAB code. Data were then pre-processed according to standard methodology prior to FC analysis (Fox et al., 2005). Data were motion corrected, spatially smoothed (5 mm) and temporally band-pass filtered (0.009 < Hz < 0.08). Further potential confound signals were removed using multiple linear regression, these included: the six motion parameters of head rotation and translation, white matter and CSF signals and the global signal, calculated by averaging the BOLD time-series across all brain voxels. FC strength was then calculated for each pairwise combination of RSN nodes as the correlation coefficient (Pearson’s *R*-value) between the mean ROI BOLD time series of each DAN, DMN, and SN node. Correlation coefficients were then converted to a normal distribution using Fisher’s *r*- to-z Transform (*z* = 0.5 Ln [(1 + *r*)/(1 - *r*)]) (Jenkins and Watts, 1968). These values were converted into *z*-scores by dividing by the square root of the variance [1/√(*n* - 3)], where *n* is the degrees of freedom in the measurement, i.e., (number of volumes-2). All negative correlations were replaced with 0 in order to address the fact that negative correlations may have been artificially induced following global signal regression (Murphy et al., 2009).

### Functional Connectivity Measures

In order to reduce the number of pairwise comparisons, we calculated composite intra- and inter-network FC for each participant. Intra-network measures consisted of averaging the FC strengths of each pair of nodes within a network. Inter-network FC strengths (ACC-DAN, ACC-DMN, right insula-DAN, right insula-DMN, PCC-DAN, PCC-SN) were calculated by averaging the FC strengths between the main node of each network, as identified previously (Seeley et al., 2007; Buckner et al., 2008, 2009), and all nodes of each other network. For example, ACC-DAN FC was calculated by averaging FC strengths across all ACC-DAN node pairs. We chose to limit our FC analysis in this way for brevity and specificity. Seeding from each node of the DMN separately would have resulted in six measures for both DMN-SN and DMN-DAN inter-network FC, making it harder to statistically compare and interpret differences between the two age groups. The main nodes we chose to focus our analysis on have all been consistently previously identified as main nodes of their corresponding networks. Average inter-network FC measures are representative of the patterns of age-group differences in FC that are seen at the individual seed-node level for each RSN. See Supplementary Material Figures S3a–c.

### Assessing Changes in Spatial Location of ROIs in Older Adults

We also assessed whether any observed differences in the strength of FC between age groups arose as a consequence of changes in the center point of the RSN nodes with older age. For this, RSN nodes were redefined for older participants using the spatial components from a separate group ICA (gICA) of only the older participant’s data. While this gICA includes fewer subjects than that used for the original node definition, it has the advantage of allowing a direct examination of the spatial location of RSN nodes in this specific group of older subjects. For node definition, the same methods were applied as for the independent cohort, except data were decomposed into 15 (rather than 20) spatially independent components with Melodic to identify RSNs most comparable to the initial analysis. Restricting this data set to 20 components resulted in the RSNs splitting into multiple components, presumably because of the smaller sample size compared to the independent cohort of younger participants used in the original analysis. Cubic ROIs were then centered on the peak voxel (as above) for each of the nodes defined from this separate gICA (**Table 1**). **Table 1** also presents the spatial differences in peak voxel location for the two methods of RSN node definition (i.e., independent cohort vs. defined specifically within the older cohort). Using these alternative ROIs we re-assessed (1) intra-network (2) ACC inter-network and (3) PCC inter-network FC in the older subjects and compared it with FC measures obtained using the young ROIs. This allowed us to investigate whether any age-related FC differences were a result of changes in FC strength or a spatial re-organization of RSN nodes in older adults.

### Statistical Analysis

IBM SPSS Statistics for Windows (Version 20.0) was used to conduct mixed design ANOVAs with the factors age and network and the interaction term age^∗^network to assess differences in intra- and inter-network FC between the two age groups. In addition, we also used this ANOVA configuration to assess whether age modulated FC differentially for the two sexes. Finally, mixed design ANOVAs with the factors gender and network and the interaction term gender^∗^network were uses to assess sex differences in FC within the two age groups. Pairwise comparisons within each ANOVA were Bonferroni corrected for multiple comparisons.

## Results

### Potential Confounds

#### Age-Related Gray Matter Volume Differences

This study explicitly attempted to address potential gray matter loss with age, which could have resulted in different proportions of gray/white matter and CSF within the ROIs for the two age groups. By segmenting each participant’s anatomical scan into the three tissue classes, and transforming partial volume maps into functional space, we were able to include only gray matter voxels in our FC analysis. This meant that for most ROIs, excluding left IPL and left OFC, older adults had, on average, significantly fewer voxels retained compared to younger adults, after correcting for multiple comparisons. In the Supplementary Material, we present the ACC-network FC results following an analysis which included all ROI voxels for both age groups (i.e., did not exclude CSF and white matter voxels) and report that the FC differences identified remain the same (Supplementary Figure S2). Therefore, any differences in ROI size between age groups are unlikely to be driving any of the FC differences we have identified.

#### Sleepiness

Another possible confound within this study is the potential for participants to fall asleep in the scanner, during the resting-state fMRI acquisition, which has previously been reported, and associated with changes in RSN organization (Tagliazucchi and Laufs, 2014). Although yet to be established, it is also possible that the propensity for sleep inside the scanner differs between younger and older adults. We found that younger and older adults did not differ in terms of daytime sleepiness, as assessed by the ESS (Younger *M* = 5.85 ± 3.48, Older *M* = 4.9 ± 4.52), or post-scan sleepiness, as assessed by the KSS (Younger *M* = 4.7 ± 1.9, Older *M* = 4.65 ± 1.81). This was revealed by a non-significant main effect of age [*F*(1,38) = 0.48, *p* = 0.49, η^2^= 0.013], and a non-significant age^∗^sleep measure interaction [*F*(1,38) = 0.44, *p* = 0.51, η^2^= 0.011]. These results suggest that, at least for this cohort, older adults were not considered more likely to fall asleep during periods of immobility, compared to younger adults.

#### Head Motion

Younger and older adults did not differ significantly in terms of relative or absolute head motion parameters as revealed by no significant main effect of age [*F*(1,38) = 0.13, *p* = 0.73, η^2^= 0.003] and no significant age^∗^motion interaction [*F*(1,38) = 0.46, *p* = 0.50, η^2^= 0.01]. Similarly, we found no effect of gender. See **Table 3** for the average head motion values for the participant groups and **Table 4** for all statistical outcomes.

**Table 3:** Average (± standard deviation) absolute and relative motion parameters (mm) for the two age groups, as a whole and divided by gender.

	Group		Male		Female	
	Young	Older	Young	Older	Young	Older
Absolute	1.43 ± 0.33	1.41 ± 0.36	1.53 ± 0.22	1.49 ± 0.35	1.30 ± 0.42	1.34 ± 0.38
Relative	0.08 ± 0.04	0.13 ± 0.07	0.08 ± 0.03	0.14 ± 0.05	0.07 ± 0.04	0.13 ± 0.08

*No significant differences were identified between the participant groups.*

**Table 4:** Outcome of mixed ANOVAs testing differences in motion (absolute and relative) for participants divided by age and gender.

		*F*	*p*	η^2^
Young	Male vs. female	2.476	0.133	0.12
	Motion ^∗^ gender	2.27	0.149	0.11
Older	Male vs. female	1.241	0.280	0.06
	Motion ^∗^ gender	0.639	0.435	0.03
Male	Young vs. old	0.046	0.832	0.003
	Motion ^∗^ age	0.626	0.439	0.03
Female	Young vs. old	0.283	0.601	0.02
	Motion ^∗^ age	0.013	0.910	0.001

*ANOVAs consisted of the main effects motion (absolute and relative) and either gender or age and the interaction terms motion^∗^gender or motion^∗^age group. F, F-statistic; p, *p*-value; η^*2*^, partial eta squared (effect size).*

### Functional Connectivity

Results are first presented for the original node definitions produced from the independent younger cohort, followed by those obtained from the older participants. All significant age and gender effects on FC can be found in **Table 5**.

**Table 5:** Significant statistical effects of age (Younger Y vs. Older O) and gender on intra- and inter-network FC measures, following FC *analysi*s using ^1^node definitions defined from the younger, independent cohort, and ^2^nodes defined separately for the older adults.

		*F*	*P*	η^2^
**Intra-network FC (group)**				
Main effect: Age^1^	Y > O	10.606	0.002	0.218
**Intra-network FC (male)**				
Main effect: age^1^	Y > O	8.18	0.01	0.312
**ACC inter-network FC (group)**				
Interaction: age^∗^network^1^		4.48	0.026	0.106
**Interaction: age^∗^network^2^**		7.33	0.004	0.16
	ACC-DAN (O > Y)		<0.001	0.46
	ACC-DMN (Y > O)		0.006	0.18
**ACC inter-network FC (male)**				
Interaction: age^∗^network^1^		4.55	0.028	0.202
	ACC-DAN (O > Y)		0.047	0.20
Interaction: age^∗^network^2^		4.76	0.024	0.21
	ACC-DAN (O > Y)		0.008	0.33
**ACC inter-network FC (female)**				
Interaction: age^∗^network^2^		4.17	0.042	0.19
	ACC-DAN (O > Y)		<0.001	0.60
	ACC-DMN (Y > O)		0.03	0.24

*Pairwise comparisons within each ANOVA were Bonferroni corrected for multiple comparisons.*

#### Intra-network FC

Older participants were found to have reduced intra-network FC compared to younger adults, as revealed by a significant main effect of age [*F*(1,38) = 10.606, *p* = 0.002, η^2^= 0.218]. No significant interaction between age and network was found [*F*(2,76) = 0.214, *p* = 0.808, η^2^= 0.006], suggesting that this reduction in intra-network FC with age was not specific to any one RSN. Furthermore, the intra-network FC was shown to differ significantly across networks, independent of age, as revealed by a significant main effect of network [*F*(2,76) = 7.921, *p* = 0.001, η^2^= 0.172]. The SN was found to have significantly greater intra-network FC in comparison to the DAN and DMN (*p* = 0.016 and *p* = 0.002), respectively. From herein, although significant main effects of network are discussed in the text for completeness, they are not highlighted on the figures, in order to clearly present any effect of age or gender. Significant network effects are expected when comparing measures of intra- and inter-network FC (e.g., ACC-SN vs. ACC-DAN) as the intra-network FC strength will typically be much greater than any measure of inter-network FC. However, as this is not a finding related to age or gender we choose not to highlight it in on the figures for clarity.

#### Intra-network FC: Sex Differences

##### Male vs. female: By age

**FIGURE 3:**
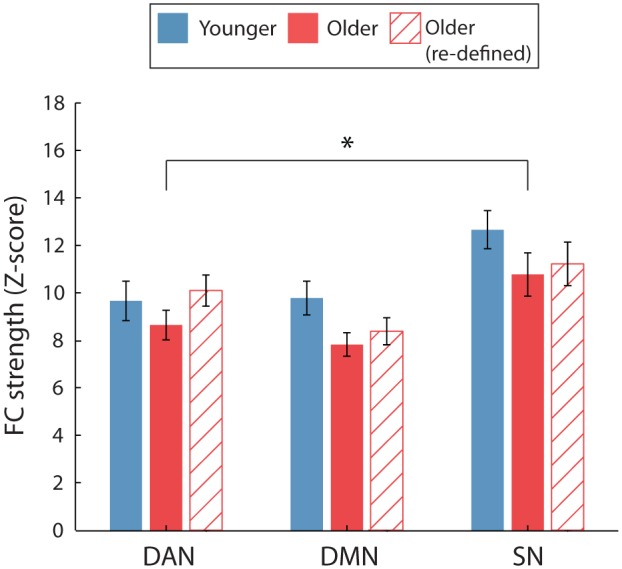
**Average intra-network FC for the two age groups.** For FC calculated using the young ROIs only (solid bars), a significant main effect of age identified that intra-network FC was weaker for older, compared to younger participants. No significant age-group difference was identified when FC was calculated using age-group specific ROIs for the older participants (hatched bars). ^∗^*p* < 0.05. Error bars are SEM calculated across participants.

We first investigated whether there were any sex differences in intra-network FC within the two age groups. For both age groups, a significant main effect of network was identified [*F*(2,36) = 3.891, *p* = 0.030, η^2^= 0.178 and *F*(2,36) = 4.280, *p* = 0.021, η^2^= 0.192, for younger and older, respectively] indicating that intra-network FC strength was dependent on the network. However, pairwise comparisons between networks failed to reach significance. For both age groups, there was no evidence to suggest that intra-network FC was significantly different between genders. This was evident by the lack of a significant main effect of gender [*F*(1,18) = 0.350, *p* = 0.562, η^2^= 0.019 and *F*(1,18) = 0.054, *p* = 0.819, η^2^= 0.003] and gender^∗^network interactions [*F*(2,36) = 1.739, *p* = 0.190, η^2^= 0.088 and *F*(2,36) = 0.440, *p* = 0.648, η^2^= 0.024]. See **Figure 3** for intra-network FC for the two age groups.

##### Younger vs. older: By gender

We then sought to identify whether age differentially modulated intra-network FC differences for the two sexes. For males, intra-network FC was significantly reduced in older, compared to younger, participants, as indicated by a main effect of age [*F*(1,18) = 8.18, *p* = 0.01, η^2^= 0.312]. No significant age^∗^network interaction was identified [*F*(2,36) = 0.209, *p* = 0.812, η^2^= 0.011], indicating that in male participants age affected intra-network FC in a global manner, rather than on a network-specific level.

**FIGURE 4:**
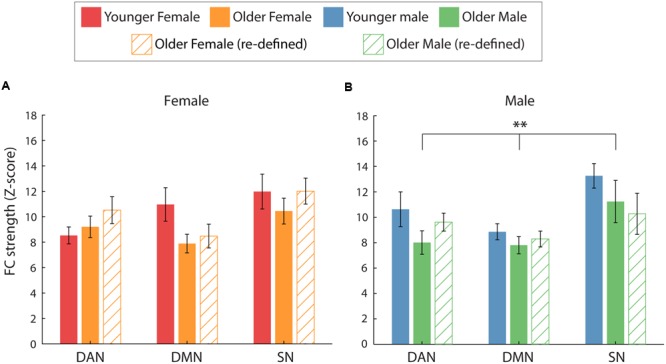
**Average intra-network FC for the two age groups (solid = young, hatch = old), split by gender. (A,B)** The effect of age on intra-network FC separately for female **(A)** and male **(B)** participants. When comparing FC using the young ROIs (solid bars), a significant main effect of age was identified for male participants only (^∗∗^*p* = 0.01). However, no significant differences between the age groups were identified when FC was calculated using age-group specific ROIs for the older participants (hatched bars).

For the females, age did not modulate intra-network FC and nor was there a significant difference in intra-network FC strength across the three networks, as revealed by the lack of a significant main effect of age [*F*(1,18) = 2.863, *p* = 0.108, η^2^= 0.137] or a significant interaction of age^∗^network [*F*(2,36) = 1.66, *p* = 0.205, η^2^= 0.084]. See **Figures 4A,B** for intra-network FC for the two sexes, divided by age group.

#### Inter-network FC

As it was unclear which node would be considered the main node of the DAN, we focused our inter-network FC analysis on the main nodes of the SN (ACC, right AI) and DMN (PCC), which have been identified previously (Seeley et al., 2007; Buckner et al., 2008, 2009).

#### ACC Inter-network FC

**FIGURE 5:**
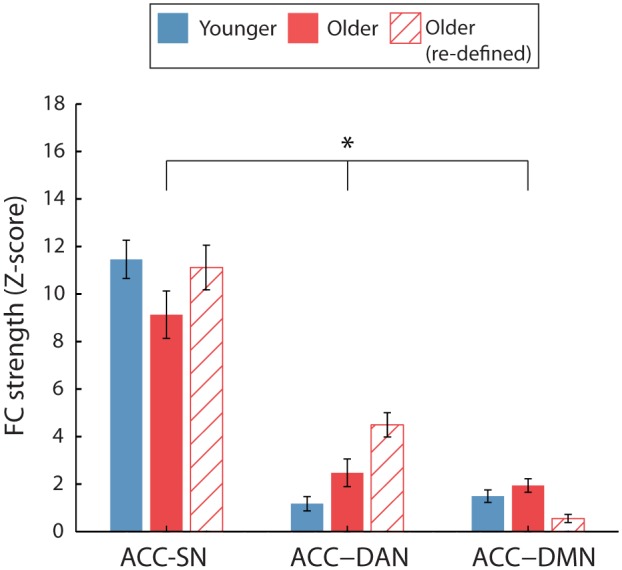
**Average ACC inter-network FC for the two age groups.** Inter-network FC is calculated by averaging FC between ACC and each node of the target network. For FC calculated using the young ROIs only (solid bars), a significant age^∗^network interaction was identified (^∗^*p* < 0.05). No significant age-group difference was identified when FC was calculated using age-group specific ROIs for the older participants (hatched bars). Error bars are SEM calculated across participants.

As expected, ACC-SN FC was significantly greater than ACC-DAN and ACC-DMN FC, as indicated by a significant main effect of network [*F*(2,76) = 120.71, *p* < 0.001, η^2^= 0.761] and pairwise comparisons between ACC-SN and ACC-DAN (*p* < 0.001) and ACC-SN and ACC-DMN (*p* < 0.001). Overall, inter-network FC was not significantly affected by age, as shown by a non-significant main effect of age [*F*(1,38) = 0.18, *p* = 0.676, η^2^= 0.005]. A significant interaction effect showed that ACC FC was differentially affected by age, depending on the network [*F*(2,76) = 4.48, *p* = 0.026, η^2^= 0.106], with a trend for reduced ACC-SN FC and increased ACC-DAN FC in older adults (**Figure 5**). However, tests of simple effects revealed that taken independently, ACC-SN, ACC-DAN and ACC-DMN FC were not significantly different for the two age groups (*p* = 0.078, η^2^= 0.08 and *p* = 0.055, η^2^= 0.093 and *p* = 0.250, η^2^= 0.035, respectively).

#### ACC Inter-network FC: Sex Differences

**FIGURE 6:**
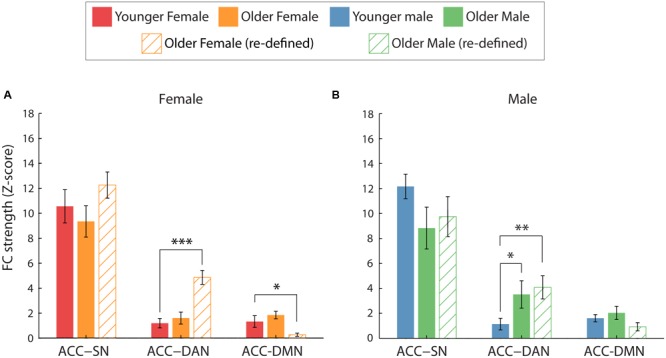
**Average ACC inter-network FC for the two age groups, split by gender. (A,B)** The effect of age on ACC-network FC for female **(A)** and male **(B)** participants. When comparing FC using the young ROIs (solid bars), greater ACC-DAN FC was identified for older, compared to younger, male participants only (^∗^*p* < 0.05). However, when FC was calculated using age-group specific ROIs (hatched bars), greater ACC-DAN FC was also identified for older, compared to younger, females (*p* < 0.001). In addition, weaker ACC-DMN FC was found for older, compared to younger, females using this method. Error bars are SEM calculated across participants.

All analyses that investigated sex differences in ACC inter-network FC revealed that ACC-SN FC was significantly greater than ACC-DAN (*p* < 0.001) and ACC-DMN (*p* < 0.001) FC, regardless of age-group or gender (**Figure 6**).

##### Male vs. female: By age

Within both age groups, male and female participants did not exhibit significantly different ACC-inter network FC. This was shown by the lack of significant main effects of gender [*F*(1,18) = 1.273, *p* = 0.274, η^2^= 0.066 and *F*(1,18) = 0.501, *p* = 0.488, η^2^= 0.027; for younger and older participants, respectively] and gender^∗^network interactions [*F*(2,36) = 0.620, *p* = 0.475, η^2^= 0.033 and *F*(2,36) = 0.755, *p* = 0.445, η^2^= 0.04; for younger and older participants, respectively].

##### Younger vs. older: By gender

For female participants, ACC inter-network FC was not significantly different between younger and older participants (**Figure 6A**). This was shown by a non-significant main effect of age [*F*(1,18) = 0.129, *p* = 0.885, η^2^= 0.001] and age^∗^network interaction [*F*(2,36) = 4.67, *p* = 0.526, η^2^= 0.035].

For male participants, ACC inter-network FC was modulated by age. We identified no general effect of age [*F*(1,18) = 0.07, *p* = 0.794, η^2^= 0.004]. However, we found that ACC inter-network FC was differentially affected by age, as shown by a significant age^∗^network interaction [*F*(2,36) = 4.55, *p* = 0.028, η^2^= 0.202]. Analysis of simple effects revealed that older men had significantly greater ACC-DAN FC compared to younger men (*p* = 0.047, η^2^= 0.202, **Figure 6B**). No significant differences in FC with age were identified for ACC-SN or ACC-DMN (*p* = 0.09, η^2^= 0.151 and *p* = 0.469, η^2^= 0.02, respectively).

#### Right AI Inter-network FC

As expected, right AI-SN FC was significantly greater than right AI-DAN or right AI-DMN FC, as indicated by a significant effect of network [*F*(2,76) = 292.11, *p* ≤ 0.001, η^2^= 0.885] and pairwise comparisons between right AI-SN and right AI-DAN and right AI-DMN (*p* < 0.001 and *p* < 0.001, respectively). Age did not have a significant impact on right AI inter-network FC, as shown by a non-significant main effect [*F*(1,38) = 0.746, *p* = 0.393, η^2^= 0.019] and a non-significant network^∗^age interaction [*F*(2,76) = 1.578, *p* = 0.218, η^2^= 0.04]. See Supplementary Figure S4 for Right AI-network FC.

#### Right AI Inter-network FC: Sex Differences

All analyses that investigated sex differences in right AI inter-network FC revealed that right AI-SN FC was significantly greater than right AI-DAN (*p* < 0.001) and right AI-DMN (*p* < 0.001) FC, regardless of age-group or gender. However, right AI inter-network FC was not significantly different between male and female participants within age groups or between age groups within the two sexes (Supplementary Figures S5a,b). This was identified by the non-significant effect of age (*p* = 0.767, η^2^= 0.005 and *p* = 0.431, η^2^= 0.035 for female and male participants, respectively) and gender (*p* = 0.511, η^2^= 0.024 and *p* = 0.942, η^2^= 0.0 for younger and older participants, respectively), as well as non-significant age^∗^network (*p* = 0.676, η^2^= 0.022 and *p* = 0.378, η^2^= 0.048 for female and male participants, respectively) and gender^∗^network interactions (*p* = 0.23, η^2^= 0.079 and *p* = 0.56, η^2^= 0.025 for younger and older participants, respectively).

#### PCC Inter-network FC

As expected, PCC-DMN FC was significantly greater than PCC-DAN and PCC-SN inter-network FC, as shown by a significant main effect of network [*F*(2,76) = 413.955, *p* < 0.001, η^2^= 0.916] and pairwise comparisons between PCC-DMN and PCC-DAN and PCC-DMN (*p* < 0.001 and *p* < 0.001, respectively). PCC inter-network FC was not significantly different between the two age groups, as indicated by a non-significant main effect of age [*F*(1,38) = 3.468, *p* = 0.07] and age^∗^network interaction [*F*(2,76) = 1.731, *p* = 0.191, η^2^= 0.044]. See Supplementary Figure S6 for PCC-network FC.

#### PCC Inter-network FC: Sex Differences

Similarly, all analyses that investigated sex differences in PCC inter-network FC identified that PCC-DMN FC was significantly greater than PCC-DAN (*p* < 0.001) and PCC-SN (*p* < 0.001) regardless of age or gender. However, PCC inter-network FC was not significantly different for male and female participants within age groups and neither did age differentially affect PCC inter-network FC for the two sexes (Supplementary Figures S7a,b). This was identified by the non-significant effect of gender (*p* = 0.476, η^2^= 0.029 and *p* = 0.563, η^2^= 0.019 for younger and older participants, respectively) and age (*p* = 0.200, η^2^= 0.089 and *p* = 0.166, η^2^= 0.104 for female and male participants, respectively), as well as non-significant gender^∗^network (*p* = 0.382, η^2^= 0.052 and *p* = 0.684, η^2^= 0.021 for younger and older participants, respectively) and age^∗^network (*p* = 0.251, η^2^= 0.074 and *p* = 0.566, η^2^= 0.031 for female and male participants, respectively) interactions.

### Spatial Re-organization of RSN Nodes in Older Adults

Qualitative comparison of the locations of the RSN nodes’ peak Z-statistic voxels between the two age groups suggests that the center of a number of the RSN nodes may shift with age. This was particularly apparent for left and right OFC, AI, and ACC (**Figure 2**). Using the ROIs defined specifically for the older adults, we re-evaluated RSN intra-network FC and inter-network FC of two main RSN nodes (ACC, PCC) (see below). We identified that by calculating older FC using the age-group specific ROIS, the differential effect of age on the two genders was attenuated. This suggests that the spatial re-organization of RSN nodes may be gender specific, in addition to the gender specific effects on FC strength, as seen above for the original node definitions. Specific results are presented below.

#### Intra-network FC

Following the re-definition of the older RSN nodes, we found no significant intra-network FC differences between age groups, as indicated by a non-significant main effect of age and a non-significant age^∗^network interaction [*F*(1,38) = 1.73, *p* = 0.20, η^2^= 0.04 and *F*(2,76) = 1.03, *p* = 0.36, η^2^= 0.026, respectively]. These results diverged from previous findings (**Figure 3**) that older adults exhibited significantly weaker intra-network FC, obtained when using the same ROIs for both age groups.

Similarly, the previous finding which suggested that the reduction in intra-network FC was specific to male participants was not fully replicated (**Figure 4**). Although a similar trend was identified for male participants, intra-network FC did not differ significantly between the age groups for either female or male participants. This was indicated by a non-significant main effect of age [*F*(1,18) = 0.20, *p* = 0.88, η^2^= 0.001 and *F*(1,18) = 4.04, *p* = 0.06, η^2^= 0.18] and age^∗^network interactions [*F*(2,36) = 2.57, *p* = 0.09, η^2^= 0.13 and *F*(2,36) = 0.66, *p* = 0.52, η^2^= 0.04], for female and male participants, respectively.

#### ACC Inter-network FC

Following the re-definition of the older RSN nodes, we report greater ACC-DAN FC in older, compared to younger, adults as previously identified (**Figure 5**). This was confirmed by a significant age^∗^network interaction [*F*(1.43,54.3) = 7.33, *p* = 0.004, η^2^= 0.16] and a significant pairwise comparison for ACC-DAN [*p* < 0.001, η^2^= 0.46], we also identified weaker ACC-DMN (*p* = 0.006, η^2^= 0.18) FC for older, compared to younger adults. ACC-SN FC did not differ significantly between the two age groups (*p* = 0.8, η^2^= 0.002).

However, greater ACC-DAN FC was no longer specific to male participants as was previously identified (**Figure 6**). Significant age^∗^network interactions were identified for both female and male participants [*F*(1.33,23.91) = 4.17, *p* = 0.04, η^2^ = 0.19 and *F*(1.52,27.41) = 4.76, *p* = 0.024, η^2^= 0.21, respectively]. Pairwise comparisons revealed that both female and male older participants exhibited greater ACC-DAN FC, compared to their younger counterparts (*p* < 0.001, η^2^= 0.60 and *p* = 0.008, η^2^= 0.33, respectively). Older female participants exhibited significantly weaker ACC-DMN FC compared to younger female participants (*p* = 0.03, η^2^= 0.24), while both younger and older male participants exhibited similar levels of ACC-DMN FC (*p* = 0.134, η^2^= 0.12). ACC-SN FC did not differ significantly between the two age groups for both female (*p* = 0.32, η^2^= 0.05) and male (*p* = 0.20, η^2^= 0.09) participants.

#### PCC Inter-network FC

As was previously identified, PCC inter-network FC was not significantly different between the two age groups (Supplementary Figure S6), as indicated by a non-significant main effect of age [*F*(1,38) = 2,58, *p* = 0.116, η^2^= 0.06] and age^∗^network interaction [*F*(1.25,47.46) = 0.30, *p* = 0.64, η^2^= 0.008].

Similarly, in correspondence with the previous results, age was not found to differentially affect PCC inter-network FC for the two sexes (Supplementary Figure S7). This was identified by non-significant main effects of age [*F*(1,18) = 1.55, *p* = 0.23, η^2^= 0.08 and *F*(1,18) = 1.32, *p* = 0.27, η^2^= 0.07] and non-significant age^∗^network interactions [*F*(1.20,21.66) = 0.16, *p* = 0.74, η^2^= 0.009 and *F*(1.35,24.74) = 0.16, *p* = 0.77, η^2^= 0.009] for both female and male participants, respectively.

## Discussion

We investigated the influence of both age and gender on intra- and inter-network FC. We identified that, when using the same RSN node definitions for the two age groups, older adults were found to have reduced intra-network FC, particularly in the DMN, and increased ACC-DAN inter-network FC in comparison to younger participants. Upon further investigation, we identified that the increased inter-network FC in older age was driven specifically by the male participants in our sample. Additional evaluation of these FC differences, using RSN node definitions that were specific to the older cohort, suggested that there was a gender-specific spatial re-organization of some RSN nodes, which were predominantly frontal in location. While greater ACC-DAN FC was still identified in older, compared to younger, participants the difference was exhibited by both genders rather than being specific to male participants. This suggests that the node definitions provided by the independent younger cohort remained appropriate for older males, but not for older females.

Our findings are largely in agreement with previous studies which have identified that advancing age is associated with reductions in the modularity of RSNs. While younger brains are typically highly modular, with limited inter-network FC (Achard and Bullmore, 2007; Bullmore and Sporns, 2012), older brains are seen to have weaker intra-network FC and stronger inter-network FC (Voss et al., 2010; Betzel et al., 2014; Geerligs et al., 2014a,b). Increased inter-network FC associated with older age is often compared to a typical finding from task-based fMRI studies, where older adults exhibit ‘over recruitment’ of brain regions during tasks, compared to younger adults (Reuter-Lorenz et al., 2000; Cabeza, 2002; Buckner, 2004; Dennis et al., 2008; Park and Reuter-Lorenz, 2009). This is commonly regarded as a compensatory mechanism to maintain or improve function (Cabeza et al., 2002; Buckner, 2004; Park and Reuter-Lorenz, 2009). However, it remains to be determined whether (1) additional inter-network FC is a compensatory mechanism which is beneficial for brain function in response to the typical age-related reduction of intra-network FC; or (2) a reduction in RSN specificity results in interference between network activity, which is not conducive to efficient task performance and may underlie less efficient processing sometimes seen in older adults (Baltes and Lindenberger, 1997). A series of studies by Geerligs et al. (2014c,d) suggested that although increased inter-network connectivity during tasks may be compensatory, older adults reach a ‘resource ceiling’ which results in age-related deficits in performance in more difficult conditions. However, they also reported that although reductions in intra-network FC were associated with poorer performance on cognitive tasks, additional inter-network FC was not always associated with better performance (Geerligs et al., 2014a). It is apparent that more research is required to fully understand the phenomenon of increased BOLD response during tasks or increased inter-network FC in older adults and to establish whether particular inter-network FC patterns are more or less beneficial than others. The relationship between increased task responses and changes to FC also remains to be clarified, both in younger and older subjects (Mennes et al., 2010, 2011).

Relatively few studies have investigated the interaction between age and gender on intra- and inter-network FC changes with age for multiple networks. Biswal et al. (2010) revealed that both age and gender were significant determinants of FC while Zuo et al. (2010) identified homotopic FC (FC between any pair of symmetric interhemispheric voxels) differences dependent on both age and gender. Similarly, (Agcaoglu et al., 2015) have shown that age can differentially affect RSN lateralization for the two sexes, while Allen et al. (2011) and Scheinost et al. (2014) also provided evidence to suggest that men and women exhibit differential patterns of FC change with age. Although our initial results suggested that changes to FC strength with age were associated with gender, further investigation suggested that the spatial organization of RSN nodes was differentially affected by age for the two sexes. For example, greater ACC-DAN FC for older, compared to younger, male participants was identified using both RSN node definition methods. However, this was only seen when using age group-specific RSN nodes for female participants, suggesting that the spatial extent and peak location of specific RSN nodes differs between male and female older participants (**Figure 2**). Age related ACC-DAN FC strength was no longer modulated by gender after applying age-group specific RSN node definitions, suggesting gender specific differences in the spatial definition of RSN nodes. Future work should look to investigate the interaction between age and gender on the re-organization of RSNs. With larger sample sizes, it would be interesting to investigate FC from gender specific RSN nodes defined separately for each age group. One caveat of the secondary analysis presented here is that the gICA used to create specific older adult RSN nodes only contained 20 participants compared to the independent cohort of 55 participants, which was used to define the RSN nodes originally, which might lead to reduced reliability of RSN definition. However, it is not clear how this could lead to the observed gender differences in older subjects. Future work should look to validate the node definition we present here to confirm the finding that RSN nodes may be spatially reorganized with older age.

One important observation is that nodes of a particular network were differentially affected by age and gender, particularly when considering inter-network FC. This was investigated specifically for the SN-DAN, and identified that ACC-DAN FC was specifically disrupted with older age, rather than a whole network re-organization of the SN (**Figures 5** and **6**; Supplementary Figures S3a,b). The fact that the increase in ACC FC was found to be specific to nodes of the DAN, rather than a global increase of ACC FC to all nodes of the RSNs investigated, suggests that this shift in ACC FC may serve to support a function. The ACC is known to modulate responses in sensory/motor/association cortices (Crottaz-Herbette and Menon, 2006; Sridharan et al., 2008; Menon and Uddin, 2010) and is strongly implicated in a wide array of cognitive processes (Koski and Paus, 2000; Shackman et al., 2011; Stevens et al., 2011). This is arguably due to the dense and diverse connectivity of the ACC (Margulies et al., 2007; Seeley et al., 2007; Shackman et al., 2011; Yu et al., 2011) which is known to have direct connections to the spinal cord (Morecraft and Tanji, 2009), and connections to subcortical, sensorimotor, cognitive, salience, pain, and affective networks (Vogt, 2005; Dosenbach et al., 2007; Margulies et al., 2007; Yu et al., 2011). This dense connectivity profile suggests that the ACC is a hub that is well situated to integrate information from multiple areas and regulate action/behavior (Shackman et al., 2011). For the same reasons, the ACC may also be well positioned to facilitate compensatory connectivity in response to disruption of FC with age. However, as previously discussed, we must also consider the possibility that such network re-organization with age is detrimental to brain function, and it remains to be seen whether increased ACC-DAN FC is associated with better or poorer cognitive performance. Similarly, Hoffstaedter et al. (2014) provided evidence that nodes of the same network can be differentially affected by age. This should be considered carefully when combining measures of FC and when investigating inter-network FC. The underlying reasons behind age-related changes in the FC of some nodes, while others are unaffected, remains unclear. In this study, we identified that for both age groups, left-right insula FC were the most connected nodes of the SN, meaning that the least functionally connected node (ACC) of a network was found to be modulated by age. Further work could look to identify whether this effect also occurs in other RSNs and to what extent it is behaviorally relevant.

We have identified that, in addition to intra-network FC, there is further information to be gleaned from studying inter-network FC and from considering the FC of individual network nodes. Individual network nodes appear to differ in their inter-network connectivity, as well as how they are affected by age and gender, which is something that is not well understood. Only by studying more RSNs in this fashion, potentially with higher dimensionality of regions (Shirer et al., 2011) and incorporating measures of cognition, may we be able to build a more coherent picture of what happens to brain connectivity as a result of the aging process. We also provide evidence that in addition to changes in FC between RSN nodes, the spatial location of certain RSN nodes may change with advancing age, the extent of which may be differentially affected by gender. These results highlight the problematic nature of comparing RSN FC changes between groups. For example, if the spatial locations of RSN nodes alter with age or disease, applying the same RSN node definition to all participants may result in spurious differences in FC strength between groups. Spatial re-organization was RSN node specific, highlighting the importance of investigating individual RSN nodes to identify which are most prone to spatial re-organization with advancing age or neurological disorder. Future work should further investigate the spatial re-organization of RSN nodes with age and utilize larger data sets to create reliable age or patient group specific RSN node definitions which can be made publically available for researchers investigating FC.

## Author Contributions

AG, RW, JH, and IP: Data acquisition and analysis. AB, SM, AG, and IP: Substantial contributions to the conception or design of the work. AB, SM, and AG: Interpretation of data and drafting of the manuscript. AB, AG, IP, JH, RW, and SM: critical revision of the manuscript for important intellectual content. All authors approves the final version of the work and agree to be accountable for all aspects of the work in ensuring that questions related to the accuracy or integrity of any part of the work are appropriately investigated and resolved.

## Conflict of Interest Statement

The authors declare that the research was conducted in the absence of any commercial or financial relationships that could be construed as a potential conflict of interest.

## Footnotes

1 www.fmrib.ox.ac.uk/fsl
