# Supplementary material for: Gender Specific Re-organization of Resting-State Networks in Older Age
Source: Front Aging Neurosci. 2016 Nov 25;8:285. doi: 10.3389/fnagi.2016.00285 (PMC5122714; doi:10.3389/fnagi.2016.00285)
Supplement: Supplementary file 1 [file Data_Sheet_1.pdf]

FC of sensory RSNs

Intra- sensory network FC

a.

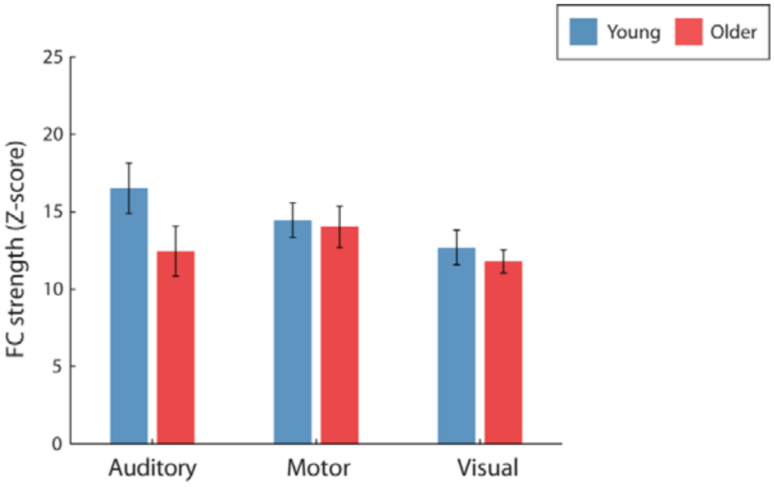

Figure S1a: Average intra-sensory network FC for the two age groups for sensory RSNs. Intra-network FC is calculated by averaging FC between each pair of nodes within the network. Error bars are SEM calculated across participants. No significant differences were identified. The RSNs consisted of the following nodes: **Auditory**: left and right superior temporal gyrus, **Motor**: left and right M1, supplementary motor area, **Visual:** left and right V1, left and right lateral visual regions.

Inter- sensory network FC

b. c.

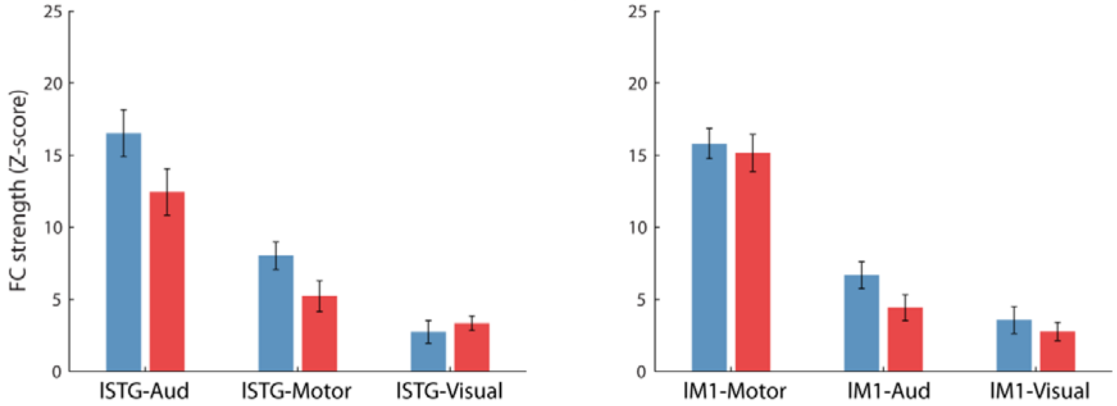

d.

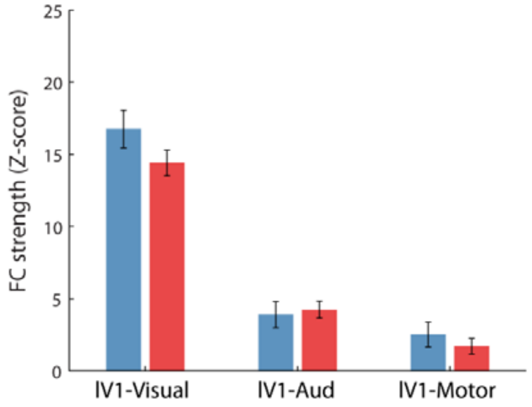

Figure S1b: Average sensory inter-network FC for the two age groups. Sensory inter-network FC is calculated by averaging FC between a seed node of each sensory network and all other nodes of the sensory RSNs. Left STG **(S1b)**, left M1 **(S1c)** and left V1 **(S1d)** were used as seed nodes to calculate auditory, motor and visual inter-network FC, respectively. Error bars are SEM calculated across participants. No significant differences were identified. Here, node definitions created using the independent younger sample were used for both age groups.

The effect of ROI size on age-related FC differences

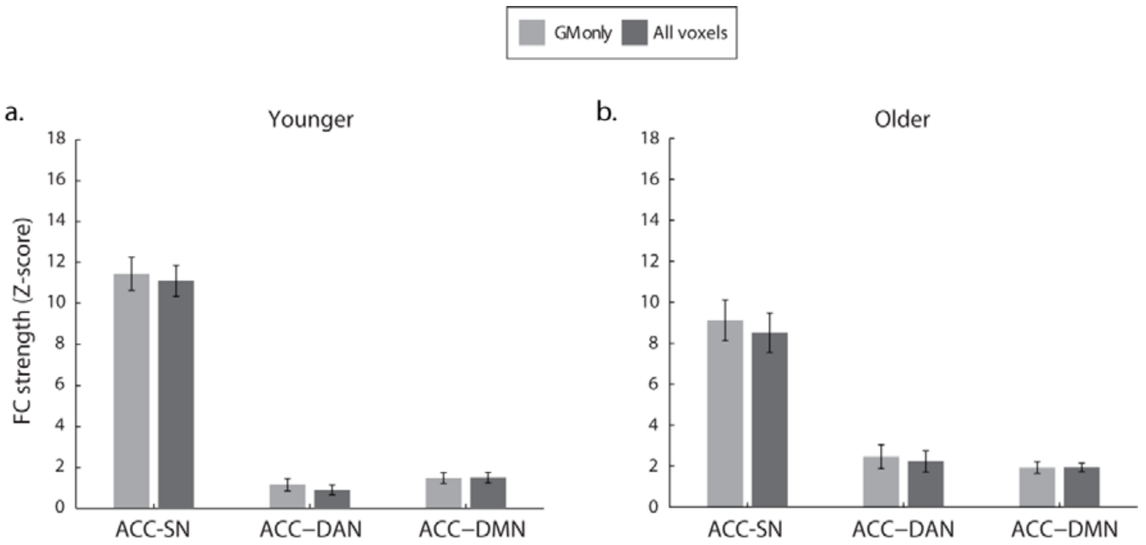

A comparison of the average ACC inter-network FC for younger (**S2a**) and older (**S2b**) participants calculated by 1) restricting FC analysis to grey matter voxels only (light grey) and 2) including all ROI voxels (dark grey) for analysis. No significant differences were identified.

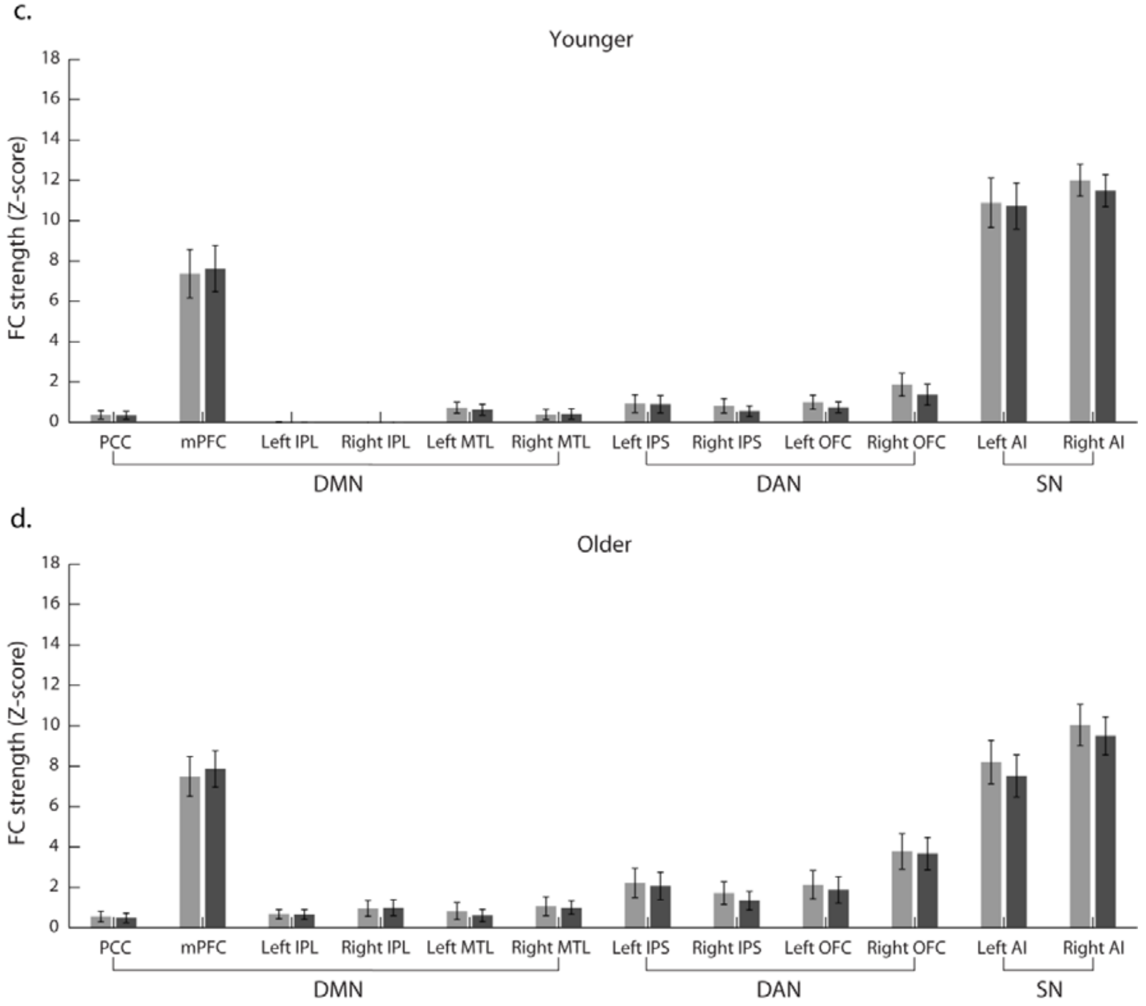

A comparison of FC between ACC and all other nodes of DMN, DAN and SN ACC for younger (**S2c**) and older (**S2d**) participants, calculated by either restricting FC analysis to grey matter voxels only (light grey) or including all ROI voxels (dark grey) for analysis. These results indicate that average inter-network FC measures (Figures S2a & S2b) are representative of the patterns of FC that are seen at the individual node level for each RSN. No significant differences were identified.

FC by individual node

Here we present the FC between the main nodes used for inter-network FC analysis, and all other RSN nodes. FC strengths were averaged across the individual nodes within a network, to create the composite scores presented in the manuscript. Figures S3a, S3b and S3c show FC between each of the seed regions (ACC, right AI and PCC respectively) and all other individual nodes of each network. These figures illustrate that the average inter-network FC measures (Figures 5, S4, S6) are representative of the patterns of age-group differences in FC that are seen at the individual node level for each RSN. For example, the average inter-network FC from the ACC increases with age with the DAN (Figure 5), and the same pattern is seen for the individual nodes that comprise the DAN (Figure S3a).

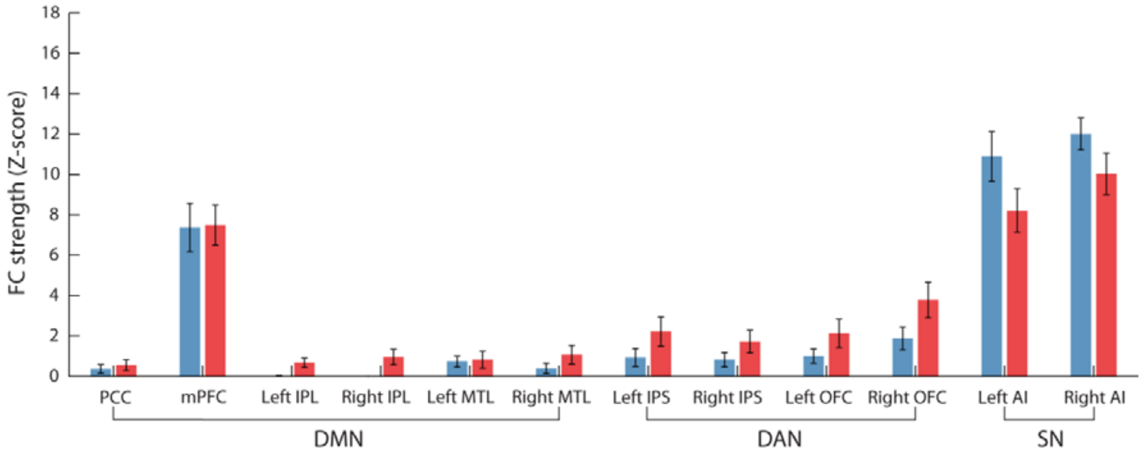

Figure S3a: FC between ACC and all other nodes of DMN, DAN and SN, using the same RSN node definitions for the two age groups. Error bars are SEM calculated across participants.

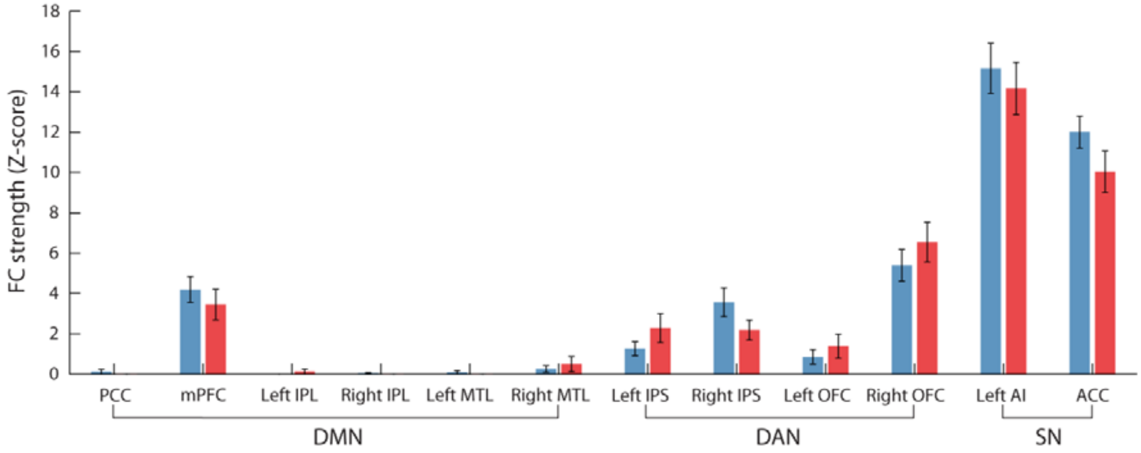

Figure S3b: FC between right AI and all other nodes of DMN, DAN and SN. Error bars are SEM calculated across participants.

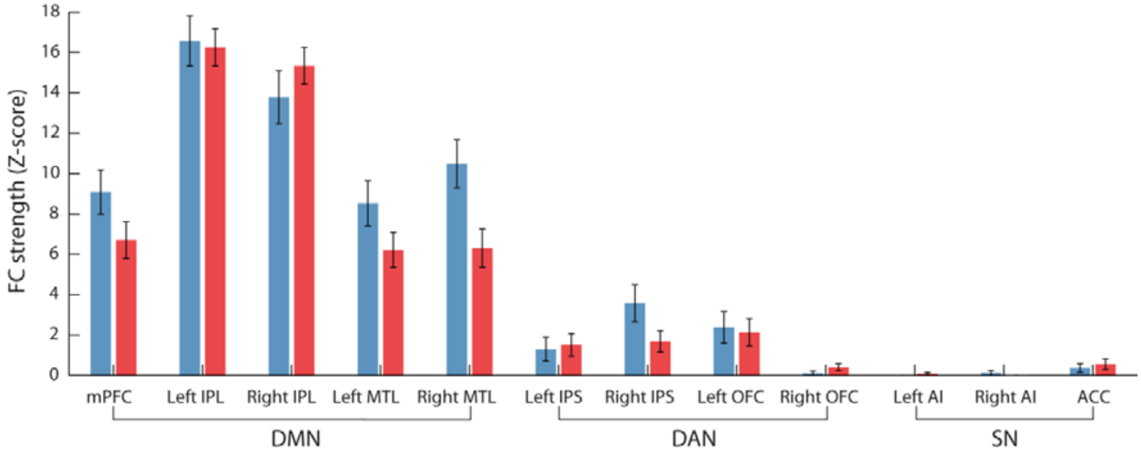

Figure S3c: FC between PCC and all other nodes of DMN, DAN and SN. Error bars are SEM calculated across participants.

Non-significant effects

Right AI inter-network FC

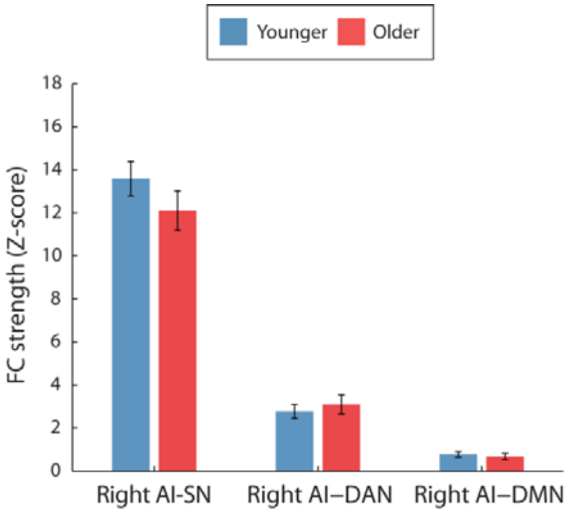

Figure S4: Average right AI inter-network FC for the two age groups. Inter-network FC is calculated by averaging FC between ACC and each node of the target network. Here, FC is calculated using the young ROIs only. No significant differences were identified. Error bars are SEM calculated across participants.

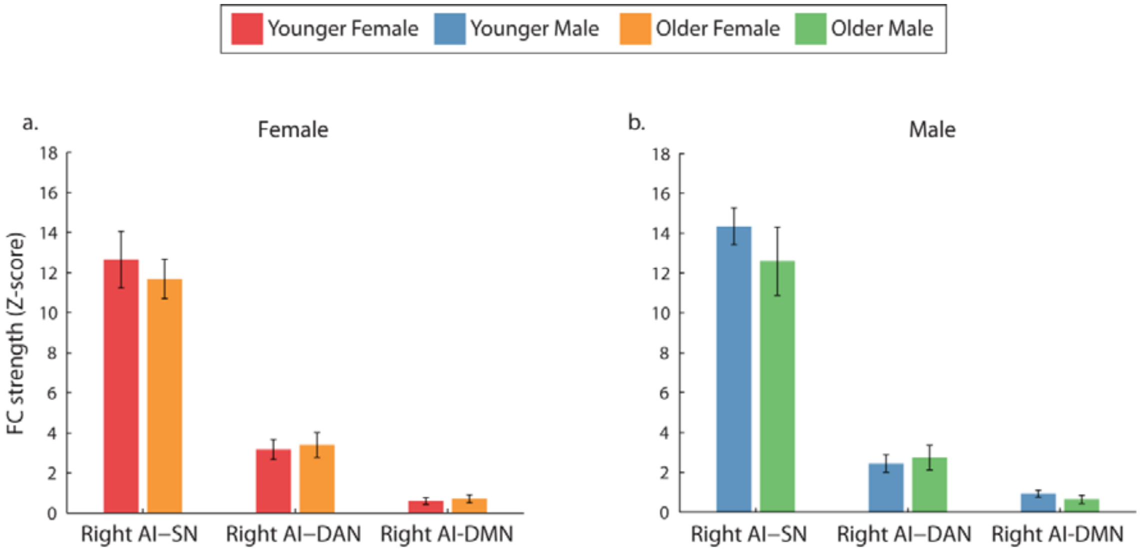

Figure S5: Average right AI-network FC for the two age groups, split by gender. Figures S5a and S5b depict the effect of age on right AI-FC for female (**S5a**) and male (**S5b**) participants. No significant differences were identified. Error bars are SEM calculated across participants.

PCC inter-network FC

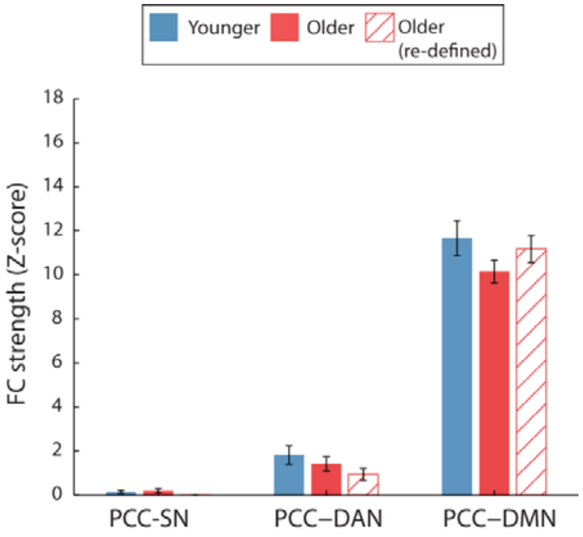

Figure S6: Average PCC inter-network FC for the two age groups, calculated using the young ROIs only (solid bars) and age-group specific ROIs for the older participants (hatched bars). No significant differences were identified using either method to define RSN nodes. Error bars are SEM calculated across participants.

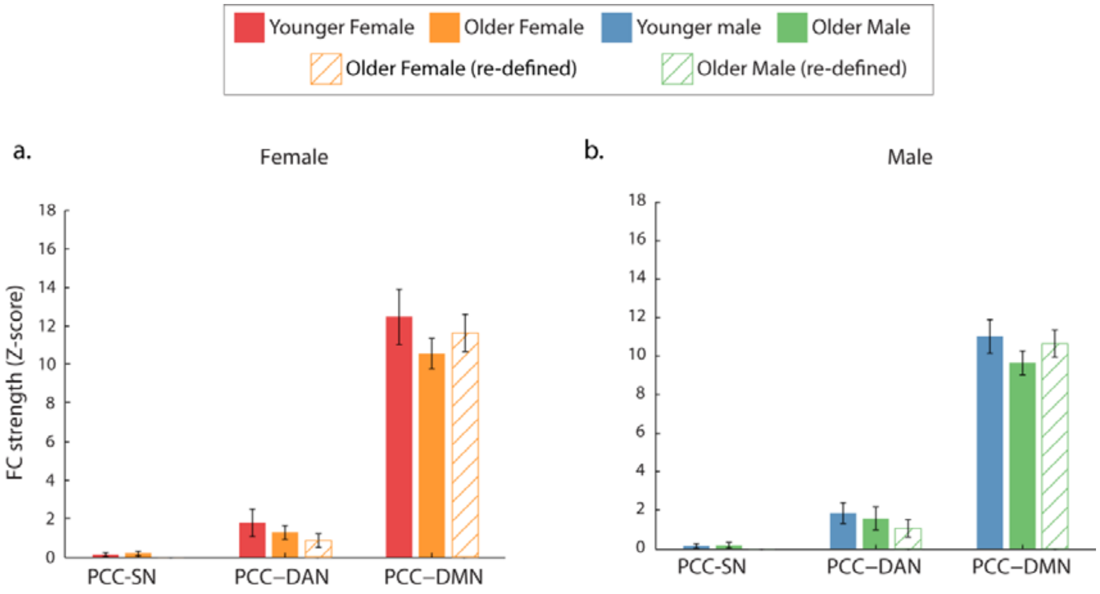

Figure S7: Figures S7a and S7b depict the effect of age on PCC-FC for female (**S7a**) and male (**S7b**) participants. No significant differences were identified using either ROIs defined from a young cohort (solid bars) or age-group specific ROIs for the older participants (hatched bars). Error bars are SEM calculated across participants.
